# Variation in branchial expression among *insulin-like growth-factor binding proteins* (*igfbps*) during Atlantic salmon smoltification and seawater exposure

**DOI:** 10.1186/s12899-017-0028-5

**Published:** 2017-01-18

**Authors:** Jason P. Breves, Chelsea K. Fujimoto, Silas K. Phipps-Costin, Ingibjörg E. Einarsdottir, Björn Thrandur Björnsson, Stephen D. McCormick

**Affiliations:** 1 Department of Biology, Skidmore College, 815 N Broadway, Saratoga Springs, 12866 NY USA; 2 Fish Endocrinology Laboratory, Department of Biological and Environmental Sciences, University of Gothenburg, Box 463, SE-40530 Gothenburg, Sweden; 3 USGS, Leetown Science Center, S.O. Conte Anadromous Fish Research Center, P.O. Box 796, One Migratory Way, Turners Falls, 01376 MA USA

**Keywords:** Insulin-like growth-factor, Binding proteins, Growth hormone, Atlantic salmon, Smoltification, Osmoregulation, Gill, Liver, Salinity

## Abstract

**Background:**

In preparation for migration from freshwater to marine habitats, Atlantic salmon (*Salmo salar* L.) undergo smoltification, a transformation that includes the acquisition of hyposmoregulatory capacity. The growth hormone (Gh)/insulin-like growth-factor (Igf) axis promotes the development of branchial ionoregulatory functions that underlie ion secretion. Igfs interact with a suite of Igf binding proteins (Igfbps) that modulate hormone activity. In Atlantic salmon smolts, *igfbp4*,−*5a*,−*5b1*,−*5b2*,−*6b1* and*−6b2* transcripts are highly expressed in gill. We measured mRNA levels of branchial and hepatic *igfbps* during smoltification (March, April, and May), desmoltification (July) and following seawater (SW) exposure in March and May. We also characterized parallel changes in a broad suite of osmoregulatory (branchial Na^+^/K^+^-ATPase (Nka) activity, *Na* ^*+*^ */K* ^*+*^ */2Cl* ^*−*^ *cotransporter 1* (*nkcc1*) and *cystic fibrosis transmembrane regulator 1* (*cftr1*) transcription) and endocrine (plasma Gh and Igf1) parameters.

**Results:**

Indicative of smoltification, we observed increased branchial Nka activity, *nkcc1* and *cftr1* transcription in May. Branchial *igfbp6b1* and *-6b2* expression increased coincidentally with smoltification. Following a SW challenge in March, *igfbp6b1* showed increased expression while *igfbp6b2* exhibited diminished expression. *igfbp5a,−5b1* and*−5b2* mRNA levels did not change during smolting, but each had lower levels following a SW exposure in March.

**Conclusions:**

Salmonids express an especially large suite of *igfbps*. Our data suggest that dynamic expression of particular *igfbps* accompanies smoltification and SW challenges; thus, transcriptional control of *igfbps* may provide a mechanism for the local modulation of Igf activity in salmon gill.

## Background

Anadromous fishes such as Atlantic salmon (*Salmo salar* L.) exhibit a life history strategy that includes an initial phase in fresh water (FW) followed by migration to marine environments [1]. The transformation of stream-dwelling ‘parr’ to seawater (SW) tolerant ‘smolts’ entails the orchestrated development of physiological, morphological and behavioral traits that support migration to, and subsequent survival in, pelagic marine environments. While dependent upon reaching a necessary size, the timing of this transformation in Atlantic salmon is initiated by environmental cues such as photoperiod and temperature [2, 3]. Depending upon latitude, this transformation typically occurs at 1–4 years of age in wild Atlantic salmon [4, 5]. The months preceding migration are termed ‘smoltification’ and this stage remains incomplete prior to downstream migration; animals of this stage are termed ‘pre-smolts’. At the peak of smoltification, salmon smolts will move downstream into estuaries and then quickly enter marine environments. Upon reaching sexual maturity in the ocean, adults use olfactory cues to return to their natal FW streams to spawn [6]. Smolts that do not gain entry into marine environments will reverse some of the acquired phenotypes such as salinity tolerance, and revert to pre-smolt phenotypes that are better suited for FW environments.

In order to sustain hydromineral balance upon entry into marine environments the parr-smolt transformation is inextricably linked with the acquisition of SW tolerance. As is the paradigm for strictly marine teleosts, the ability of smolts to inhabit SW is sustained by an array of solute- and water-transporting activities in the gill, gut, kidney and urinary bladder [7]. Inasmuch as the gill is the primary tissue for the active transport of monovalent ions, the recruitment of branchial ionocytes (SW-type ionocytes) that extrude Na^+^ and Cl^−^ is essential for gaining SW tolerance. SW-type ionocytes employ ion pumps, cotransporters and channels such as Na^+^/K^+^-ATPase (Nka), Na^+^/K^+^/2Cl^−^ cotransporter 1 (Nkcc1) and cystic fibrosis transmembrane regulator 1 (Cftr1) [8, 9]. Accordingly, branchial Nka activity peaks concomitantly with *nkcc1* and *cftr1* mRNA levels when salmon achieve maximal SW tolerance [10–12]. Thus, the seasonal patterns of these three parameters reliably predict whether juvenile salmon will be able to sustain hydromineral balance upon exposure to SW [11, 12].

In Atlantic salmon, several endocrine systems synchronize the ontogeny of osmoregulatory systems with downstream migration [1, 12]. In particular, the growth hormone (Gh)/insulin-like growth factor (Igf) axis exhibits enhanced activity at the peak of smoltification [1, 13]. From the perspective of whole-organism performance, a connection between the somatotropic axis and salmonid osmoregulation is supported by numerous findings of improved salinity tolerance following exogenous Gh and/or Igf1 treatment [13–15]. The hyposmoregulatory actions of Gh are seemingly mediated by multiple molecular pathways, including: 1) branchial Gh receptors (Ghr1), 2) the synthesis and secretion of Igf1 from the liver, 3) the local production of Igf1 in the gill, and/or 4) enhanced responsiveness to cortisol [16–21]. Irrespective of the route of action, Gh and Igf1 promote salinity tolerance by regulating branchial Nka activity [22], the gene and protein expression of ionoregulatory factors [8, 23] and ionocyte density [24, 25].

Igfs interact with cognate binding proteins termed Igf binding proteins (Igfbps). The coordinated production, both spatially and temporally, of Igfbps permits modulation of Igf bioavailability in both positive and negative fashions [26]. Igfbps may also exert ligand-independent activities [27]. Studies on teleost Igfbps have primarily focused upon how they mediate growth responses to stressors such as food restriction, temperature, hypoxia, and handling [28], while relatively few studies have investigated Igfbp responses to ionoregulatory challenges [29–32]. Attributed to multiple whole-genome duplication events, Atlantic salmon express an expansive set of 19 *igfbp* genes [33]. Among these *igfbps*, *igfbp4*,−*5a*,−*5b1*,−*5b2*,−*6b1* and*−6b2* are highly expressed in the gill [33]. There is currently no understating of how branchial *igfbp* expression is modulated in preparation for ionoregulatory challenges faced by developing salmon.

Given that parr-smolt transformation encompasses numerous physiological preparations underlying marine survival, and consequently recruitment, knowledge of its physiological control informs efforts aimed at restoring endangered populations [34, 35]. Thus, the physiology of Atlantic salmon smoltification presents an important physiological context for how Igfbps underlie Gh/Igf-mediated life-history transitions. In turn, our first objective was to assess whether *igfbp* mRNA levels change during smoltification. We further investigated whether *igfbps* respond to abrupt transfer to SW, and whether such responses varied with the degree of SW tolerance. Because the gill is a key tissue underlying the development of SW adaptability, we primarily focused on *igfbp* transcripts that exhibit significant branchial expression.

## Methods

### Animals

Atlantic salmon (*Salmo salar*) parr were obtained in October of 2013 from the Kensington National Fish Hatchery, Kensington, CT, and held at the Conte Anadromous Fish Research Center, Turners Falls, MA. Individuals from this cohort were expected to smolt in the spring of 2014 on the basis of their size (>12 cm fork length) in early February [36]. Fish were held in a 1.5 m diameter fiberglass tank supplied with dechlorinated tap water under natural photoperiod. Water temperature was maintained at 9 °C until late June; water was then maintained at 10.5 °C until the conclusion of the experiment. Fish were fed to satiation twice daily with commercial feed (Bio-Oregon, Longview, WA). All experiments were carried out in accordance with US Geological Survey institutional guidelines and an approved IACUC review (SP 9065).

### Experimental design

To sample juvenile Atlantic salmon before, during, and after smolting, fish (*n* = 8) of mixed sex were sacrificed on March 3, April 8, May 1 and July 10 at 09:00 h (Eastern Standard Time), with food withheld for 24 h prior to sampling. In addition, SW challenges were conducted on March 3 and May 1 at 09:00 h. Sixteen smolts were transferred to a tank with recirculating SW (35 ppt) at 9 °C with particle and charcoal filtration and continuous aeration. Food was withheld for the duration of the challenge. Fish were sampled (*n* = 8) at 09:00 h at 24 and 48 h after transfer to SW.

### Sampling

At the time of sampling, fish were netted and anesthetized in buffered MS-222 (100 mg/l; pH 7.0; Sigma, St. Louis, MO). Blood was collected from the caudal vasculature by a needle and syringe treated with ammonium heparin. Blood samples were collected within 5 min from the initial netting. Blood was separated by centrifugation at 4 °C and plasma stored at−80 °C until analyses. Body mass and fork length were measured for calculation of condition factor: (body mass, g)/(fork length, cm)^3^ × 100. Gill and liver tissues were collected and immediately frozen on dry ice and stored at−80 °C. Four to six additional gill filaments were placed in ice-cold SEI buffer (150 mM sucrose, 10 mM EDTA, 50 mM imidazole, pH 7.3) and stored at−80 °C.

### Plasma and gill analyses

Plasma Gh levels were measured by a radioimmunoassay (RIA) validated for Atlantic salmon by Björnsson et al. [37]. Plasma Igf1 levels were measured by a RIA validated for salmonids [38]. Plasma chloride was analyzed by the silver titration method using a Buchler-Cotlove digital chloridometer (Labconco, Kansas City, MO) and external standards. Branchial Nka activity was determined as described by McCormick [39]. Protein concentration of the gill homogenate was determined using a BCA protein assay (Thermo Fisher Scientific, Rockford, IL).

### RNA extraction, cDNA synthesis and quantitative real-time PCR (qRT-PCR)

**Fig. 1:**
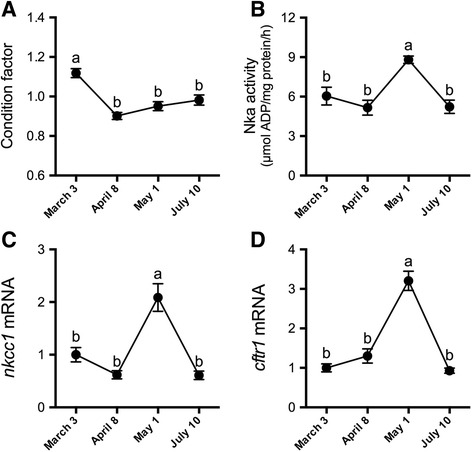
Seasonal dynamics of condition factor and ionoregulatory parameters. Condition factor **(a**) and branchial Nka activity **(b**), *nkcc1* (**c**) and *cftr1* (**d**) mRNA levels in Atlantic salmon maintained in FW from March 3 through July 10. Means ± S.E.M. (*n* = 8). mRNA levels are presented as a fold-change from the March 3 group. Means not sharing the same letter are significantly different (one-way ANOVA, Tukey’s HSD test, *P* < 0.05)

**Fig. 2:**
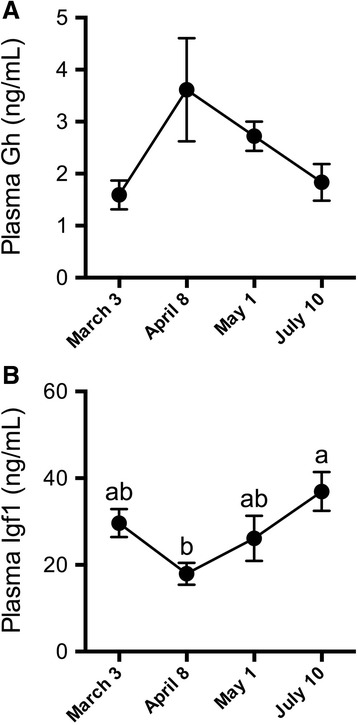
Seasonal dynamics of plasma hormones. Plasma Gh (**a**) and Igf1 (**b**) levels in Atlantic salmon maintained in FW from March 3 through July 10. Means ± S.E.M. (*n* = 8). Means not sharing the same letter are significantly different (one-way ANOVA, Tukey’s HSD test, *P* < 0.05)

**Fig. 3:**
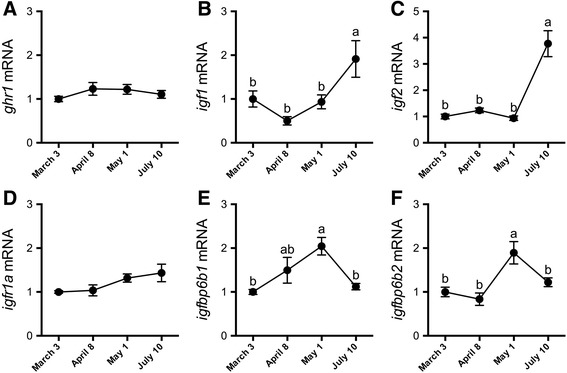
Seasonal dynamics of branchial gene expression. Branchial *ghr1* (**a**), *igf1* (**b**), *igf2* (**c**), *igfr1a* (**d**), *igfbp6b1* (**e**) and -*6b2* (**f**) mRNA levels in Atlantic salmon maintained in FW from March 3 through July 10. Means ± S.E.M. (*n* = 8). mRNA levels are presented as a fold-change from the March 3 group. Means not sharing the same letter are significantly different (one-way ANOVA, Tukey’s HSD test, *P* < 0.05)

**Fig. 4:**
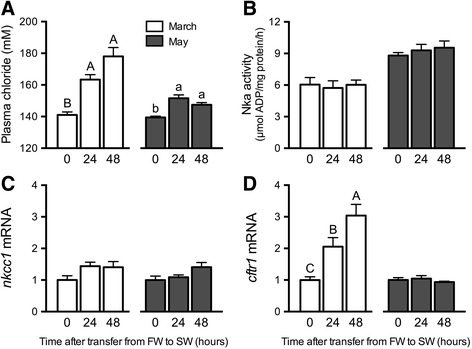
Effects of SW exposure on ionoregulatory parameters. Plasma chloride (**a**) and branchial Nka activity (**b**), *nkcc1* (**c**) and *cftr1* (**d**) mRNA levels in Atlantic salmon subjected to 24 and 48 h SW exposures in March (open bars) and May (shaded bars). Means ± S.E.M. (*n* = 8). mRNA levels are presented as a fold-change from the 0 h groups. Within a given experiment, denoted by uppercase or lowercase letters, means not sharing the same letter are significantly different (one-way ANOVA, Tukey’s HSD test, *P* < 0.05)

**Fig. 5:**
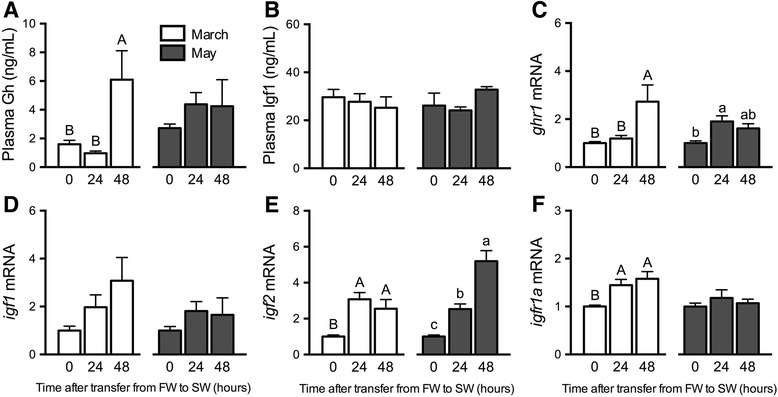
Effects of SW exposure on plasma hormones and branchial gene expression. Plasma Gh (**a**), Igf1 (**b**), and branchial *ghr1* (**c**), *igf1* (**d**)*, igf2* (**e**) and *igfr1a* (**f**) mRNA levels in Atlantic salmon exposed to 24 and 48 h SW exposures in March (open bars) and May (shaded bars). Means ± S.E.M. (*n* = 8). Within a given experiment, denoted by uppercase or lowercase letters, means not sharing the same letter are significantly different (one-way ANOVA, Tukey’s HSD test, *P* < 0.05)

**Fig. 6:**
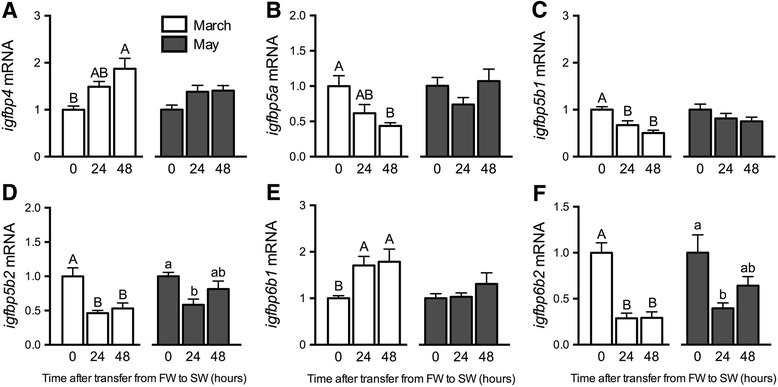
Effects of SW exposure on *igfbp* gene expression. Branchial *igfbp4* (**a**),−*5a* (**b**),−*5b1* (**c**),−*5b2* (**d**),−*6b1* (**e**) and−*6b2* (**f**) mRNA levels in Atlantic salmon exposed to 24 and 48 h SW exposures in March (open bars) and May (shaded bars). Means ± S.E.M. (*n* = 8). Within a given experiment, denoted by uppercase or lowercase letters, means not sharing the same letter are significantly different (one-way ANOVA, Tukey’s HSD test, *P* < 0.05)

Total RNA was extracted from tissues by the TRI Reagent procedure (MRC, Cincinnati, OH) according to the manufacturer’s protocols. RNA concentration and purity were assessed by spectrophotometric absorbance (Nanodrop 1000, Thermo Scientific, Wilmington, DE). First strand cDNA was synthesized with a High Capacity cDNA Reverse Transcription Kit (Life Technologies, Carlsbad, CA). Relative mRNA levels were determined by qRT-PCR using the StepOnePlus real-time PCR system (Life Technologies). We employed previously described primer pairs for *ghr1* [23], *igf1*, *igf2*, *igf receptor 1a* (*igfr1a*) and *elongation factor 1α* (*ef1α*) [40], *igfbp1a1*,−*1b1*,−*1b2*,−*2a*,−*2b1*,−*2b2*,−*4*,−*5a*,−*5b1*,−*5b2*,−*6b1* and*−6b2* [33], and *nkcc1* and *cftr1* [11]. qRT-PCR reactions were setup in a 15 μl final reaction volume with 400 nM of each primer, 1 μl cDNA and 7.5 μl of 2× SYBR Green PCR Master Mix (Life Technologies). The following cycling parameters were employed: 10 min at 95 °C followed by 40 cycles at 95 °C for 15 s, 60 °C for 30 s and 72 °C for 30 s. After verification that levels did not vary across groups, *ef1α* levels were used to normalize target genes. Reference and target gene levels were calculated by the relative quantification method with PCR efficiency correction [41]. Standard curves were prepared from serial dilutions of gill or liver cDNA and included on each plate to calculate the PCR efficiencies for target and normalization genes (>90%). Relative mRNA levels are reported as a fold-change from the March 3 group (Figs. 1–3; Table 1) or 0 h groups (Figs. 4–6; Table 2).

**Table 1:** Branchial and hepatic mRNA levels in Atlantic salmon maintained in FW from March 3 through July 10

Target Gene	March 3	April 8	May 1	July 10
Gill				
*igfbp4*	1.00 ± 0.08^b^	1.06 ± 0.20^b^	1.49 ± 0.15^a,b^	1.61 ± 0.10^a^
*igfbp5a*	1.00 ± 0.15^a,b^	0.79 ± 0.14^b^	1.51 ± 0.18^a^	0.73 ± 0.05^b^
*igfbp5b1*	1.00 ± 0.06^a,b^	0.47 ± 0.06^c^	1.17 ± 0.14^a^	0.70 ± 0.09^b,c^
*igfbp5b2*	1.00 ± 0.12^a,b^	0.57 ± 0.07^c^	1.13 ± 0.06^a^	0.76 ± 0.05^b,c^
Liver				
*ghr1*	1.00 ± 0.30^b^	2.75 ± 0.33^a^	1.09 ± 0.16^b^	0.38 ± 0.08^b^
*igf1*	1.00 ± 0.21^b^	3.04 ± 0.71^a^	3.49 ± 0.15^a^	3.25 ± 0.35^a^
*igf2*	1.00 ± 0.27	0.91 ± 0.31	0.56 ± 0.11	0.46 ± 0.07
*igfbp1a1*	1.00 ± 0.45	0.69 ± 0.37	0.10 ± 0.04	0.02 ± 0.004
*igfbp1b1*	1.00 ± 0.21^b^	2.50 ± 0.47^a^	1.05 ± 0.14^b^	0.36 ± 0.02^b^
*igfbp1b2*	1.00 ± 0.16^b^	5.75 ± 1.26^a^	2.01 ± 0.27^b^	0.21 ± 0.04^b^
*igfbp2a*	1.00 ± 0.10	0.83 ± 0.07	0.74 ± 0.06	0.73 ± 0.07
*igfbp2b1*	1.00 ± 0.10^a^	0.51 ± 0.06^b^	0.44 ± 0.05^b^	0.44 ± 0.03^b^
*igfbp2b2*	1.00 ± 0.12	1.29 ± 0.15	0.97 ± 0.09	0.90 ± 0.02

Expression levels are presented as a fold-change from the March 3 group. Means ± S.E.M. (*n* = 8). For a given transcript, means not sharing the same superscript letter are significantly different (one-way ANOVA, Tukey’s HSD test, *P* < 0.05)

**Table 2:** Hepatic mRNA levels in Atlantic salmon exposed to 24 and 48 h SW exposures in two separate experiments (March and May)

Target Gene	Experiment	0 h	24 h	48 h
*ghr1*	March	1.00 ± 0.30^a,b^	0.36 ± 0.04^b^	1.95 ± 0.55^a^
	May	1.00 ± 0.14	5.38 ± 4.00	1.79 ± 0.25
*igf1*	March	1.00 ± 0.21	0.67 ± 0.22	0.84 ± 0.30
	May	1.00 ± 0.04^b^	0.61 ± 0.17^b^	1.55 ± 0.15^a^
*igf2*	March	1.00 ± 0.27	0.63 ± 0.14	0.72 ± 0.20
	May	1.00 ± 0.19	4.39 ± 3.35	0.95 ± 0.27
*igfbp1a1*	March	1.00 ± 0.44^b^	6.06 ± 2.81^a,b^	20.15 ± 8.67^a^
	May	1.00 ± 0.39	20.26 ± 10.23	144.97 ± 88.44
*igfbp1b1*	March	1.00 ± 0.21^b^	4.45 ± 0.39^a^	4.55 ± 1.15^a^
	May	1.00 ± 0.13	0.79 ± 0.09	1.38 ± 0.47
*igfbp1b2*	March	1.00 ± 0.16	2.41 ± 0.31	2.79 ± 0.89
	May	1.00 ± 0.14^a^	0.52 ± 0.04^b^	1.24 ± 0.21^a^
*igfbp2a*	March	1.00 ± 0.10	0.98 ± 0.22	0.84 ± 0.17
	May	1.00 ± 0.09	0.85 ± 0.11	1.27 ± 0.23
*igfbp2b1*	March	1.00 ± 0.10	0.91 ± 0.06	0.65 ± 0.12
	May	1.00 ± 0.11	1.02 ± 0.14	1.53 ± 0.32
*igfbp2b2*	March	1.00 ± 0.12	0.87 ± 0.06	0.97 ± 0.13
	May	1.00 ± 0.09	0.82 ± 0.10	0.91 ± 0.19

Expression levels are presented as a fold-change from the 0 h groups. Means ± S.E.M. (*n* = 8). Within an experiment, means not sharing the same superscript letter are significantly different (one-way ANOVA, Tukey’s HSD test, *P* < 0.05)

### Statistics

Group comparisons were performed by one-way ANOVA followed by Tukey’s HSD test. Significance for all tests was set at *P* < 0.05. All statistical analyses were performed using GraphPad Prism 6 (San Diego, CA).

## Results

### Developmental/seasonal patterns

We verified that parr-smolt transformation and subsequent loss of salinity tolerance occurred by profiling multiple morphological and ionoregulatory parameters. Condition factor was significantly reduced on April 8, May 1 and July 10 compared with March 3 (Fig. 1a). We observed progressive silvering of the body and darkening of the fin margins leading to the May 1 sampling (data not shown). Branchial Nka activity was elevated in May compared with pre-smolts (March 3 and April 8) and post-smolts (July 10) (Fig. 1b). Branchial *nkcc1* and *cftr1* levels were elevated in May compared with all other sampled time points (Fig. 1c, d).

There was a significant effect of season on plasma Gh (one-way ANOVA; *P* < 0.001), but no significant differences between sampling points were detected by *post hoc* analysis (Fig. 2a). Plasma Igf1 was elevated in July when compared with April levels (Fig. 2b).

Parr-smolt transformation did not coincide with any differences in branchial *ghr1* or *igfr1a* expression (Fig. 3a, d). Both *igf1* and−*2* were elevated in post-smolts (July 10) compared with all preceding time points (Fig. 3b, c). Branchial *igfbp6b1* and*−6b2* (Fig. 3e, f) were significantly elevated near the peak of smoltification (May 1) while there were no clear seasonal effects on *igfbp4*,−*5a*, *5b1* and*−5b2* (Table 1). In liver, *ghr1* expression was elevated in April above all other time points, while *igf1* expression was elevated above March 3 levels at all subsequent samplings. There were no clear seasonal effects on hepatic *igf2*. Hepatic *igfbp1b1* and*−1b2* were elevated in April above all other time points; *igfbp2b1* was reduced from March 3 levels at all subsequent samplings (Table 1).

### Seawater exposures in March and May

In both March and May, SW exposures elicited increases in plasma chloride at 24 and 48 h (Fig. 4a). The increase in plasma chloride after SW exposure was substantially greater in March than in May. There were no significant increases in branchial Nka activity or *nkcc1* after SW exposure in March or May (Fig. 4b, c). SW exposure induced branchial *cftr1* expression in March, but not in May (Fig. 4d).

Plasma Gh levels were elevated by 48 h after SW exposure in March; Gh levels did not respond to SW exposure in May (Fig. 5a). SW exposures did not elicit any changes in plasma Igf1 (Fig. 5b). SW induced branchial *ghr1* levels by 48 and 24 h in March and May, respectively (Fig. 5c). Branchial *igf1* was not responsive to SW in March or May (Fig. 5d) whereas SW induced *igf2* in both March and May (Fig. 5e). In March, *igfr1a* showed modest increases in response to SW (Fig. 5 f).

Among the *igfbps* expressed in the gill, *igfbp4* and−*6b1* were induced by SW exposure in March (Fig. 6A, E), while *igfbp5a*,−*5b1*,−*5b2* and−*6b2* were diminished following SW exposure (Fig. 6B-D, F). As in March, *igfbp6b2* was decreased following SW exposure in May (Fig. 6 f). In liver, there were no clear effects of SW exposures on *ghr1* and *igf2*; however, SW induced *igf1* in May. *igfbp1a1* and−*1b1* were similarly induced by SW exposure in March (Table 2).

## Discussion

The progressive increase in the salt secretory capacity of the gill during smoltification entails developmentally cued patterns of ionocyte differentiation and proliferation, in addition to altered gene transcription within these ionocytes [8, 42, 43]. Knowing that the Gh/Igf axis directs the timing and nature of these cellular behaviors [13–15], we hypothesized that Igfbps contribute to smoltification and therefore would exhibit seasonal and SW-responsive patterns of gene expression. We report for the first time that increases in *igfbp6b1* and−*6b2* expression coincided with parr-smolt transformation and multiple *igfbp4*,−*5* and−*6* isoforms were modulated following SW exposures at different stages of smolt development.

Fish in this study underwent smoltification as indicated by multiple parameters. First, we observed the typical decrease in condition factor due to changes in body shape and the utilization of lipid and glycogen stores [36, 44–47]. In strong agreement with previous studies, branchial Nka activity and *nkcc1* and *cftr1* expression concomitantly peaked in May, a hallmark of SW-type ionocyte recruitment [10, 11, 20, 36, 47, 48]. The ability of juvenile salmon to maintain ionoregulatory balance upon direct transfer from FW to SW is readily employed as an operational definition of hyposmoregulatory capacity. In March (pre-smolts) we observed relatively large increases in plasma chloride after SW exposure, whereas in May (smolts), we observed modest increases in plasma chloride after SW exposure. It is interesting that in March, when fish had not yet developed SW tolerance, only *cftr1*, and not *nkcc1*, was activated in parallel with chloride perturbations. The branchial epithelium of pre-smolts harbors a population of SW-type ionocytes that presumably employ Cftr1 and Nkcc1 in the apical and basolateral cell membrane, respectively [8, 49, 50]. Within these cells, transcription of *cftr1* may be rapidly activated by ionic/osmotic conditions (environmental or internal), a pattern reminiscent of how chloride secretion is activated in opercular epithelia of *Fundulus heteroclitus* [51]. In any event, the attendant changes in the gill with respect to ionocyte function are consistent with a developmental/seasonal increase in ion-secretion capacity.

Photoperiod-induced increases in plasma Gh levels coincide with Atlantic salmon smoltification [52] and we likewise observed a rise (albeit not significant following *post hoc* analysis) in plasma Gh levels in April. Nilsen et al. [53] observed increased plasma Gh levels in SW-challenged smolts in May, whereas we observed a Gh response in March. This Gh response was paralleled by increased branchial *ghr1* expression. Kiilerich et al. [20] similarly observed increased *ghr1* with SW transfer, albeit in smolts transferred to SW in April. While not specifically shown in salmonids yet, Gh release from the pituitary is induced by direct osmosensing in certain euryhaline species such as Mozambique tilapia (*Oreochromis mossambicus*) [54]. This mode of regulation is compatible with increased plasma Gh levels when blood plasma conditions, such as plasma chloride and presumably osmolality, were perturbed following SW transfer. The osmoregulatory actions of Gh are mediated by its capacity to increase circulating levels and local tissue production of Igfs [14]. Seasonal patterns of circulating Igf1 in Atlantic salmon smolts are variable. In some cases, increases [36, 55], decreases [53], or no well-defined changes [56] have been reported. While we did not observe increased plasma Igf1 in smolts, we did observe increased hepatic *igf1* expression, perhaps mediated by increased sensitivity to Gh via upregulation of *ghr1*. On the other hand, local *igf1* and−*2* expression in the gill was not elevated in May smolts, patterns important to consider in light of the *igfbp* responses we subsequently observed.

This is the first time branchial *igfbp*s have been assessed in a salmonid preparing for seaward migration; we assayed *igfbp4*,−*5* and−*6* isoforms that exhibit robust branchial expression [33]. *igfbp4* exhibited a steady rise in expression throughout spring and summer, with increased expression following SW exposure in March. The function of Igfbp4, at least in mammals, highly depends on the physiological context surrounding its production, and may operate as either a stimulator or inhibitor of Igf1/2 signaling [57, 58]. The activities of a teleost Igfbp4 were first assessed in fugu (*Takifugu rubripes*) where overexpression delayed embryonic development [59]. Nonetheless, in Atlantic salmon and sea bream (*Sparus aurata*), *igfbp4* expression is implicated in mediating enhanced post-prandial/fasting muscle growth [40, 60–62], thereby suggesting a stimulatory effect on Igf activity. The concomitant increases of *igfbp4* along with *igf2* and *igfr1a* following SW exposure may reflect a transcriptional program underlying enhanced paracrine signaling in response to ionoregulatory demands.

In contrast with *igfbp4*, *igfbp5a*,−*5b1* and−*5b2* were all reduced following SW exposure in March. Similar to Atlantic salmon *igfbp5s*, zebrafish (*Danio rerio*) *igfbp5a* and−*5b* are expressed in the gill [63]. *igfbp5a* is expressed in a sub-population of zebrafish ionocytes termed “NaR cells” specialized for Ca^2+^ uptake via Trpv5/6 channels. *igfbp5a* plays an essential role in Ca^2+^ homeostasis; *igfbp5a* expression is induced by low environmental [Ca^2+^] and *igfbp5a* knockdown inhibits compensatory increases in NaR cell proliferation following reductions in [Ca^2+^] [31]. While not yet established for Atlantic salmon, Ca^2+^ uptake across rainbow trout (*Oncorhynchus mykiss*) gill epithelia similarly employs a Trpv5/6 channel expressed in ionocytes and pavement cells [64]. If Igfbp5a is a conserved regulator of branchial Ca^2+^ uptake, then the SW-induced reductions in *igfbp5a* we observed in this study may reflect the increased [Ca^2+^] of SW compared with FW, and subsequent down regulation of Ca^2+^ uptake pathways. Interestingly, Dai et al. [63] showed that among the zebrafish Igfbp5 isoforms (−5a and−5b), only Igfbp5b exhibits ligand-independent transactivational activity. Thus, while *igfbp5a*,−*5b1*, and−*5b2* showed similar responses to SW in the current study, it is likely that they are functionally distinct from one another, but such distinctions are entirely unresolved to date.

Wang et al. [65] described two teleost co-orthologs of human Igfbp6, denoted Igfbp6a and−6b. *igfbp6a* exhibits low expression in both zebrafish and Atlantic salmon gill, with *igfbp6b2* being highly expressed in salmon gill [33, 65]. Among the *igfbps* we assayed, *igfbp6b1* and−*6b2* showed seasonal increases in expression with maximum levels in May smolts. Mammalian Igfbp6 exhibits a higher binding affinity for Igf2 versus Igf1, and inhibits Igf actions [66]. Similarly, zebrafish Igfbp6a and−6b attenuate Igf activities and embryonic growth and development [65]. There is currently no information on plasma Igf2 dynamics during smoltification; however, locally produced Igfbp6b1 and/or−6b2 may modulate Igf2 activity in the gill. Moreover, Igfbp6 modulates cell proliferation, migration, and apoptosis in mammalian systems [66, 67], and considering how cell turnover underlies branchial development during smoltification [42], Igfbp6s may similarly contribute to cell-cycle regulation in smolts. Adding further complexity is the disparate regulation of the two *igfbp6* isoforms following SW exposures. Nonetheless, the seasonal patterns of both *igfbp6s* suggest future study of their role(s) in the gill is warranted.

We also assayed hepatic *igfbp1* and−*2* isoforms because their translated products modulate endocrine Igfs [26]. As in mammals, *igfbp1* and−*2* isoforms are highly expressed in teleost liver [33, 68–74]. Igfbp1 inhibits somatic growth, development, and glucose metabolism by restricting Igfs from binding Igf receptors [69, 75, 76]. The only report to date of plasma Igfbp dynamics in smolts (coho salmon; *Oncorhychus kisutch)* revealed an April peak in plasma Igfbp1 [77]. This elevated Igfbp1 coincided with a drop in condition factor. Here, we observed 2.5- and 5.6-fold increases in *igfbp1b1* and -*1b2* expression, respectively, in April compared with March. Recall that we also observed a decline in condition factor in early spring, a pattern that routinely occurs when Atlantic salmon smolts, but not parr, are allowed to feed ad libitum [36]. Reduced condition factor is due to both smoltification-related changes in body shape and the utilization of energy reserves such as lipid stores and liver glycogen [44, 45, 47]. Previous work has established that Gh is involved in the lipolysis that occurs during smolting, and likely interacts with cortisol to affect other catabolic changes [78]. When these patterns are further considered with the feeding ecology of migrating smolts [46, 79, 80], smoltification emerges as inherently catabolic. Thus, *igfbp1b1* and−*1b2* may be further modulating growth and metabolism as part of the metabolic demands of smolt development and in preparation for seaward migration. Interestingly, we detected no seasonal changes in *igfbp1a1*, an isoform that is sensitive to nutrient conditions [81]. Hepatic *igfbp1a1* was, however, induced by SW exposure, a response also seen with a 32-kD Igfbp (putative Igfbp1) in rainbow trout plasma [30, 82]. Collectively, these patterns suggest that duplicated *igfbp1s* allow for multifactorial control of Igf signaling during development and in response to salinity change. Future work should investigate whether divergent *igfbp1* responses are aligned with contrasting sensitivities to hormones such as cortisol, thyroid hormones and insulin, which display seasonal changes and/or mediate stress responses [83–85].

With a subset of hepatic and branchial *igfbps* whose expression patterns parallel parr-smolt transformation now revealed, future study is warranted to resolve how these dynamics relate to changes in circulating levels of actual Igfbp proteins. As the liver is regarded as a major source of circulating Igfbp1 [75, 86], we hypothesize that plasma Igfbp1b1 and−1b2 may be enhanced in early April provided that mRNA levels are suggestive of protein production and secretion. Moreover, with marked changes in branchial *igfbp6b1* and−*6b2* levels occurring in May, it should be resolved whether their translated products are retained (and act) locally, or whether they enter circulation as endocrine factors. In any event, the development of isoform-specific detection of Igfbps is the next step towards establishing how the complex expression patterns of Atlantic salmon *igfbps* across varied tissues [33] relate to local and endocrine protein levels.

## Conclusions

Salmonids express an especially wide array of *igfbps*, and our data suggest that multiple Igfbps may contribute to the development of SW tolerance and associated metabolic changes that occur during the parr-smolt transformation. With *igfbps* such as *igfbp6b1* and−*6b2* showing increases coincident with smolt development, the challenge is to now identify the specific activities of these isoforms. By comparing the physiologies of anadromous and landlocked salmon populations, investigators have discerned how relaxed selection for SW adaptability affects both endocrine and ionoregulatory systems [11, 53, 87]. We propose that a similar approach, which compares *igfbp* expression patterns across Atlantic salmon populations, will aid our understanding how Igfbps operate within, and modulate, the hormonal mechanisms that drive smoltification.

## Abbreviations

Cftr: Cystic fibrosis transmembrane regulator
Ef1α: Elongation factor 1α
FW: Fresh water
Gh: Growth hormone
Ghr: Growth hormone receptor
Igf: Insulin-like growth-factor
Igfbp: Insulin-like growth-factor binding protein
Igfr: Insulin-like growth-factor receptor
Nka: Na^+^/K^+^-ATPase
Nkcc: Na^+^/K^+^/2Cl^−^ cotransporter
qRT-PCR: Quantitative real-time PCR
RIA: Radioimmunoassay
SW: Seawater
